# 

**Published:** 1874-01

**Authors:** 


### Books & Periodicals.

The Scientific American, heads the list of the most valuable exchanges that come to our table. More real enjoyable information is gained through the reading of its columns, than any dozen "family newspapers" that can be mentioned. Its subscription price is only $3.00 per year, to be had of the publishers, Munn & Co. 34Park Row, N. Y.

Webster's Unabridged Dictionary.—We call the attention of Medical Students and Practitioners to the fact that in this book they not only possess a medical dictionary of great completeness, but also one for all the sciences. It should be found in every physician's library. Price $12.00, to be had at Hall Bros., this city, and of book dealers everywhere ; or furnished to any one who will send us thirty subscribers to the *Bistoury*, at 50 cents each.

Peterson is always on hand. We scarcely remember the time when this excellent lady's magazine was not a regular visitor to our editorial table, where it remains just long enough for us to get a glimpse of it, when it is carried off by the female portion of our household. It is always welcome.

New Book on the Ear.—Dr. D. B. St. John Roosa has delivered himself of a new "work," "a practical treatise on the Diseases of the Ear," which he offers the medical fraternity as containing new plans of treatment for the various diseases to which the ear is liable. We have read it, we are sorry to say, as it consumed time that could have been more profitably employed. The book contains *nothing* new, it being simply a compilation from the works of a few German, English and American authors, without, in many instances, giving them proper credit for the views therein expressed. It is a sort of piratical authorship, that injures no one but the projector.

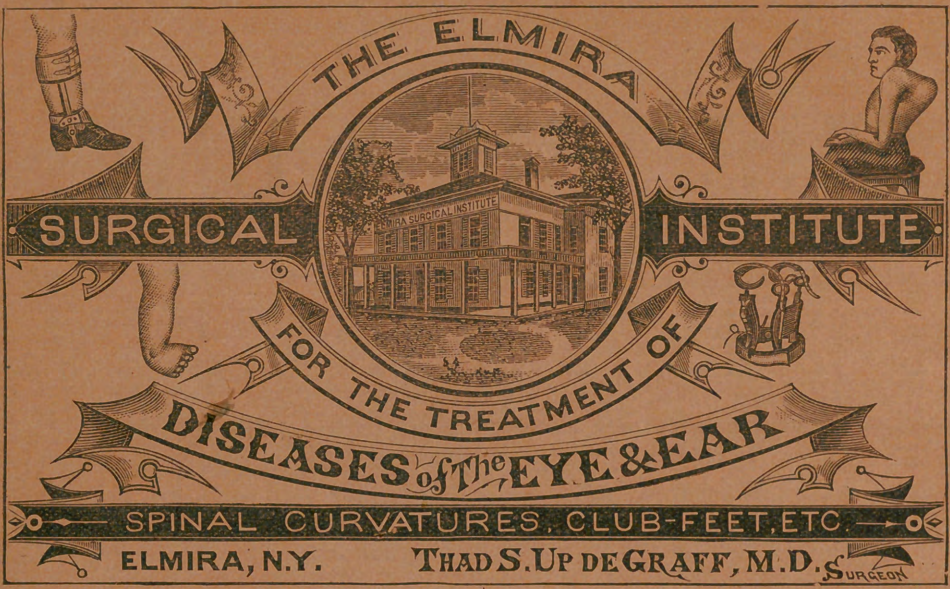

### SUBSCRIPTIONS RECEIVED.

In this column we will credit every subscription received during the last quarter. Subscribers will please notify us immediately if their payments are not duly credited. The State only is given—this, with the full name of the party subscribing, will be sufficient to indicate who is meant:

NEW YORK.—Dr. S. B. Manning, Dr. L. M. Jones, Dr. Samuel Allen, Mrs. S. M. Layton, Rev. M. L. Jones, Dr. P. Zimmer, L. P. Edwards, L. B. Johnson, J. R. Callow, V. M. Slaughter, Dr. Cummings, E. R. Eversetts, Mrs. J. L. Davidson, P. M. Josylin, Dr. O. B. House.

PENNSYLVANIA.—Dr. J. W. Sheets, Dr. O. L. Hies, Dr. S. S. Hillbish, Dr. H. I. Hottenstien, Dr. A. M. Gougler, Dr. J. P. Ziegler, Mrs. A. Whorton, Dr. A. English, L. V. Kress, J. R. Simons.

INDIANA.—Dr. James Covell, Mrs. L. Lovell.

KENTUCKY.—J. K. Harris, Dr. L. Staboll,

MISSOURI.—Dr. C. Snow, Dr. I. Kalish,

ONTARIO, Canada.—John L. Davies.

SOUTH CAROLINA.—Mrs. M. G. McAllister, Dr. O. L. Erlich.

WEST VIRGINIA.—James Otis, S. R. Waterhouse.

No P. O. Address:—Mrs. J. B. Harrison.
No Name.—Tuscumbia, Ala.

### AN ELEGANT PREMIUM!

The Young Folks' Rural, Two Chromos & The Bistoury,

ALL FOR

**ONE DOLLAR AND FIFTY CENTS.**

THE YOUNG FOLKS' RURAL is a rural and literary monthly journal, for young people of country and city. It is a beautifully printed, and elegantly illustrated sixteen page paper, brim full of entertaining stories, sketches, puzzles, plays, enigmas, charades, riddles, etc., etc., of surpassing interest to young people. We regard it as the very best youth's journal published in this country. It is published in Chicago, by H. N. F. Lewis, at $1.50 a year. Two elegant Chromos given to every subscriber.

We offer it together with Chromos and Bistoury, one year, for $1,50.

Address, **THE BISTOURY,**
ELMIRA, N. Y.

### THE BISTOURY GIVEN AWAY.

OUR TERMS FOR CLUBBING WITH OTHER JOURNALS.

We will cause any of the publications below to be sent together with The Bistoury, for one year, on the following terms:—

| | PUB. PRICE. | WITH BISTOURY. |
|---|---|---|
| American Agriculturalist | $1 50 | $1 50 |
| Scribner's Monthly | 4 00 | 4 00 |
| Peterson's Ladies' Magazine | 2 00 | 2 00 |
| Home and Health | 1 50 | 1 50 |
| Little Corporal | 1 50 | 1 50 |
| Good Health | 2 00 | 2 00 |
| Atlantic Monthly | 4 00 | 4 00 |
| Western Rural | 2 00 | 2 00 |
| Young Folks' Rural | 1 50 | 1 50 |
| Peter's Musical Monthly | 3 00 | 3 00 |
| Eclectic Magazine | 5 00 | 5 00 |
| TheGalaxy | 4 00 | 4 00 |
| Hearth and Home | 4 00 | 4 00 |
| Godey's Lady's Book | 3 00 | 3 00 |
| Wood's Household Magazine | 1 00 | 1 00 |
| The Children's Hour | 1 50 | 1 50 |
| Saturday Evening Post | 2 50 | 2 50 |
| Phrenological Journal | 3 00 | 3 00 |
| Medical Times, Philadelphia (Weekly) | 5 00 | 5 00 |
| The London Medical Record (Weekly) | 5 00 | 5 00 |
| Merry's Musem | 1 50 | 1 50 |
| Ballou's Monthly | 1 50 | 1 50 |
| Arthur's Home Magazine | 2 50 | 2 50 |
| Monthly Novelet | 2 00 | 2 00 |
| Our Young Folks | 2 00 | 2 00 |
| Ohio Farmer | 2 00 | 2 00 |
| Cultivator and Country Gentleman | 2 50 | 2 50 |
| New York Independent | 2 50 | 2 50 |
| American Union | 2 50 | 2 50 |
| Rural New Yorker | 3 00 | 3 00 |
| Saturday Night | 3 00 | 3 00 |
| Ladies' Repository | 3 50 | 3 50 |
| Harper's Magazine | 4 00 | 4 00 |
| " Bazar | 4 00 | 4 00 |
| " Weekly | 4 00 | 4 00 |
| Every Saturday | 5 00 | 5 00 |
| North American Review | 6 00 | 6 00 |
| Lippincott's Magazine | 4 00 | 4 00 |
| American Farmer's Advocate | 1 00 | 1 00 |
| Toledo Weekly Blade | 2 00 | 2 00 |

Forward the price of any of the above publications to our address, and you will receive both journals regularly, for one year. Address

**The Bistoury,** Elmira, N. Y.

### A USEFUL PREMIUM.

For one hundred and ten subscribers to THE BISTOURY, at *Fifty Cents each*, amounting to $55,00, we will give a

GROVER & BAKER SEWING MACHINE, COSTING $55 00

Everybody knows what a GROVER & BAKER Machine is, so that it requires no puffing. We will guarantee them to be new, and in perfect and complete order.

For twenty subscribers, at fifty cents each, we will send to the getter up of the club, one of

**McAllister's New Microscopes,**

The price of which is Five Dollars.

For seventy subscribers we will give as a premium a good, silver, hunting case, AMERICAN WATCH, worth $35,00.

Those who prefer to work for money, can do so according to our

### CASH LIST.

Examine it and learn *How to make from ten to twenty dollars a day!*

| | | | | |
|---|---|---|---|---|
| For 100 | Subscribers and | $50,00 | you may retain | $25,00 |
| " 50 | " | 25,00 | " " | 10,00 |
| " 25 | " | 12,50 | " " | 5,00 |
| " 10 | " | 5,00 | " " | 2,00 |
| " 5 | " | 2,50 | " " | 1,00 |

So that you get your premiums in SOLID CASH, instead of cheap books, worthless pictures, or "pinched beck" jewelry.

**THE WAY TO REMIT TO US.**

Collect $50 from 100 Subscribers. Then put $25 of it in your own pocket for your work and buy a Post Office order or draft with the other $25, and remit it to us. Also, send the names and address, (Post Office, County and State,) of each subscriber, PLAINLY WRITTEN, when they will receive THE BISTOURY by return mail, which will be an evidence to them that your contract has been faithfully performed. For fifty subscribers you will send us $15, you retaining $10, and so on, down through the list.

Women, who are worn out with sewing, throw down your needles, take a copy of THE BISTOURY and solicit subscriptions from your neighbors. You will find it a healthier and more profitable employment.

Post Masters can, without trouble, secure large lists at their offices, and make it pay better than their salaries.

Young men, out of employment, can earn a suit of clothes in one day, and make their canvassing pay them ever afterwards.

Boys and girls can canvass for THE BISTOURY,

**AND MAKE MORE MONEY**

than they ever dreamed of possessing before.

Try it, everybody. There is money in it, and

**You have your Pay in your own hands.**

Send all sums over $5 by Post Office Order or Draft, made payable to THAD S. UP DE GRAFF, M. D. Smaller sums may be sent in currency.

☞ Those who desire to get up Clubs, will have sample copies sent them FREE, by writing for them.

